# Elevated Cardiovascular Risk and Mortality in Type 2 Diabetes Patients with Cannabis, Opioid, and Cocaine Use Disorders

**DOI:** 10.1192/j.eurpsy.2026.10871

**Published:** 2026-07-31

**Authors:** S.-K. Kao, M.-J. Lu, Y.-T. Yu

**Affiliations:** 1 Psychiatry, Taipei Veterans General Hospital; 2 General Medicine, Shin Kong Wu Ho-Su Memorial Hospital; 3 Family Medicine, Landseed International Hospital, Taipei, Taiwan

## Abstract

**Introduction:**

Substance use disorders (SUDs), including those related to cannabis, opioids, and cocaine, are growing in prevalence and have been associated with adverse health outcomes, including elevated cardiovascular risk. Cardiovascular disease (CVD) remains the leading cause of morbidity and mortality in individuals with type 2 diabetes mellitus (T2DM).

**Objectives:**

This study aimed to evaluate the association between SUDs and major cardiovascular events and all-cause mortality among individuals with T2DM.

**Methods:**

Using the TriNetX Research Network, we performed a retrospective cohort analysis of adults aged ≥18 years diagnosed with T2DM between January 1, 2016, and December 31, 2022. Patients with a diagnosis of an SUD, including cannabis, opioid, or cocaine use disorders, within one year prior to T2DM diagnosis were identified. Propensity score matching was conducted to balance demographics, comorbidities, and medication use between the SUD and non-SUD cohorts.

**Results:**

After propensity score matching, there were 69,373 individuals in each group. Over a 5-year follow-up, individuals with SUDs exhibited significantly elevated risks of acute myocardial infarction (HR [95% CI] = 2.916 [2.528, 3.365], p < 0.001), atrial fibrillation (HR [95% CI] = 1.994 [1.735, 2.292], p < 0.001), heart failure (HR [95% CI] = 2.685 [2.428, 2.968], p < 0.001), and all-cause mortality (HR [95% CI] = 1.621 [1.546, 1.701], p < 0.001). Subgroup analyses demonstrated consistent risk elevation across all substance types, with the highest relative risk of mortality observed in the opioid-related disorder group (HR [95% CI] = 1.804 [1.69, 1.926], p < 0.001).

**Table 1.** Risks of AMI, HF, AF and mortality in the SUD cohort and non-SUD cohort Table 1. Long description.

Outcome	SUD (N= 69,373)	non-SUD (N= 69,373)	Hazard ratio (95% CI)	*p* value
AMI	657	263	2.916 (2.528, 3.365)	<.001*
HF	1,256	548	2.685 (2.428, 2.968)	<.001*
AF	537	314	1.994 (1.735, 2.292)	<.001*
Mortality	4,074	2,836	1.621 (1.546, 1.701)	<.001*

* Indicates statistical significance (*p* < .05). *SUD* Substance use disorder; *AMI* Acute myocardial infarction; *AF* Atrial fibrillation; *HF* Heart failure; *HR* Hazard ratio; *CI* Confidence interval.

**Fig. 1.** Cohort flowchart.

**Fig. 2.** Kaplan–Meier curves of CV events and mortality in T2DM with vs. without SUD (95% CI shaded).

**Fig. 3.** Subgroup analysis by substance type; 95% CI lower limit >1 indicates higher risk.

**Abbreviations:**

T2DM, type 2 diabetes mellitus; SUD, substance use disorder; AMI, acute myocardial infarction; AF, atrial fibrillation; HF, heart failure; HR, hazard ratio; CI, confidence interval.

**Image 1:**
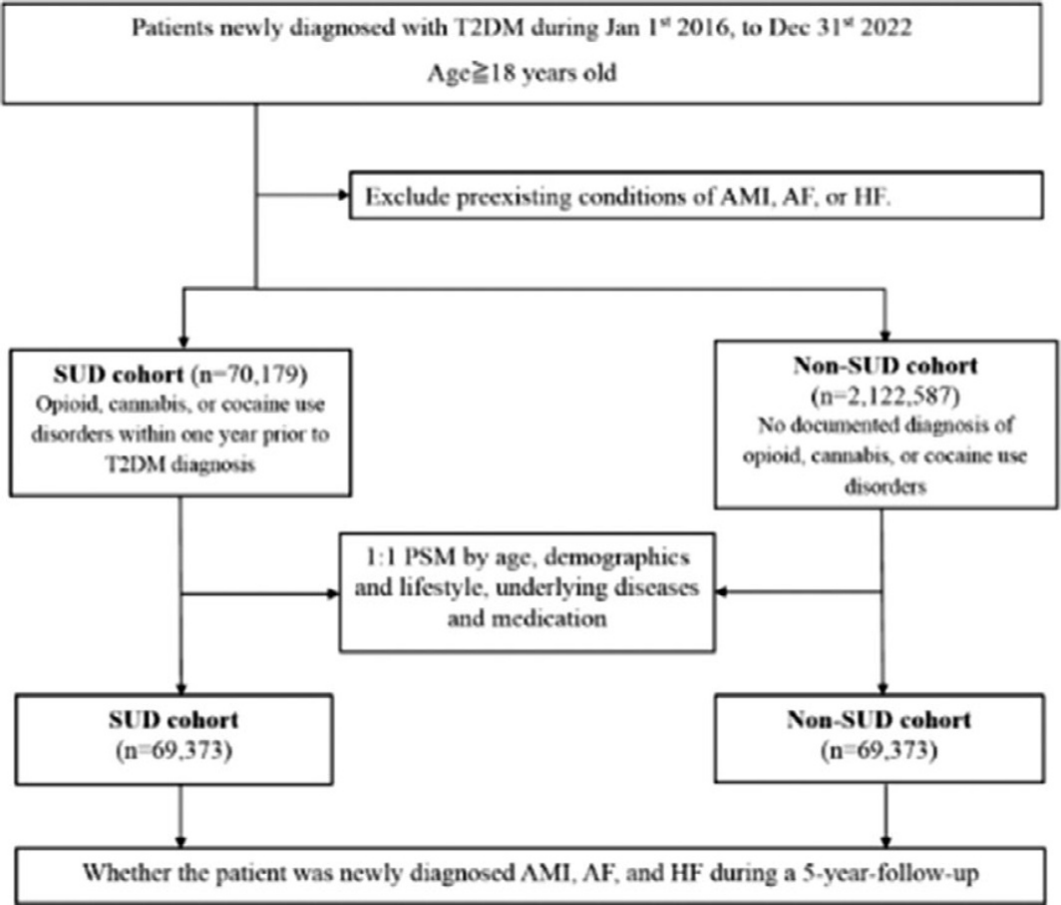
Image 1: Long description.

**Image 2:**
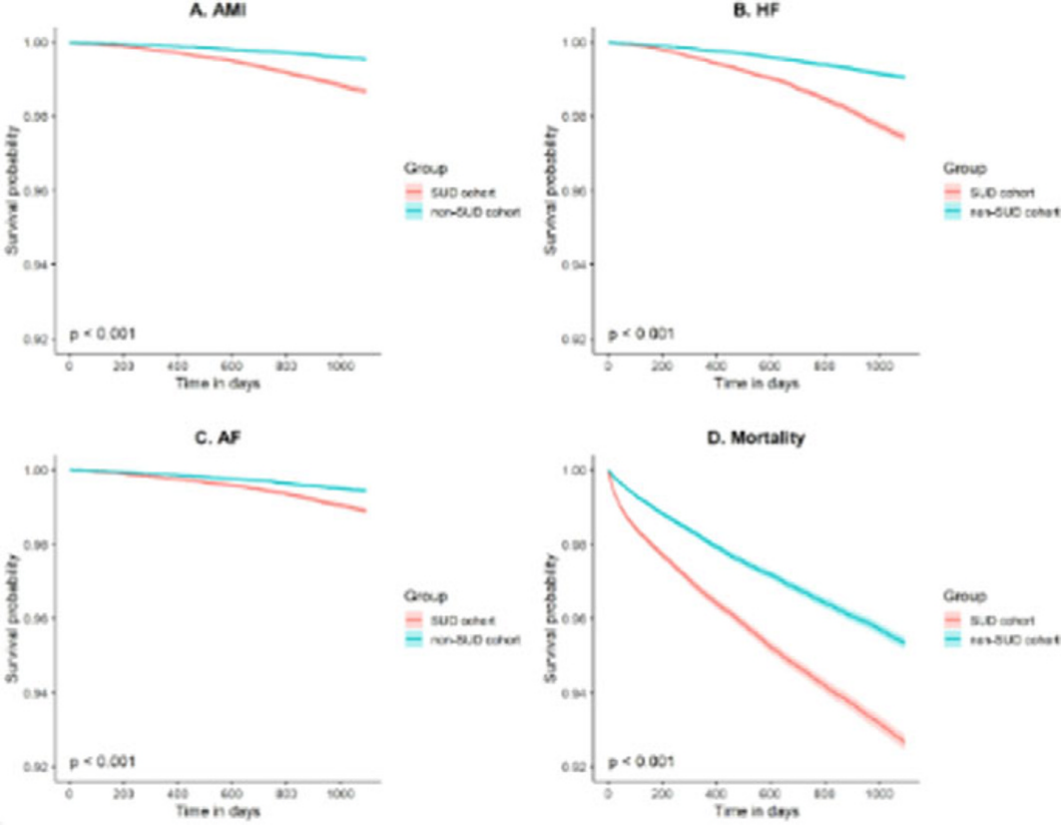
Image 2: Long description.

**Image 3:**
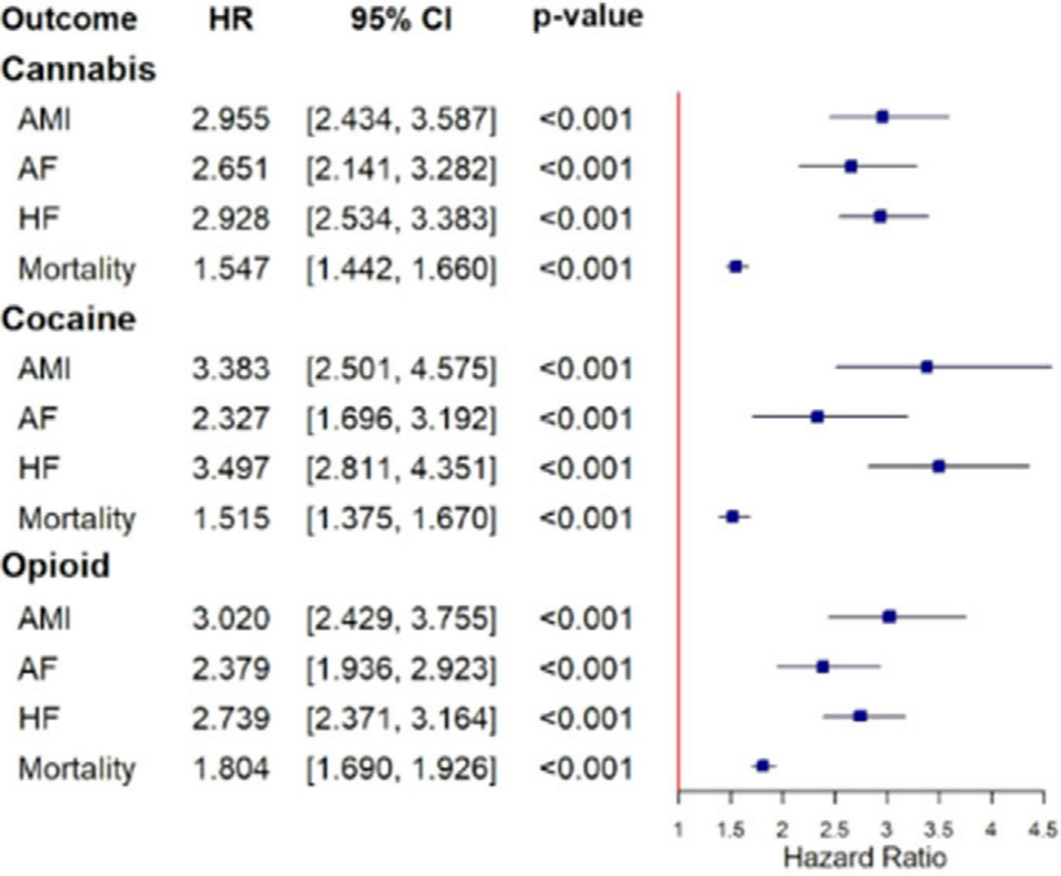
Image 3: Long description.

**Conclusions:**

The presence of an SUD is associated with a markedly increased risk of cardiovascular morbidity and all-cause mortality in individuals with T2DM. These findings underscore the need for integrated cardiovascular risk stratification and the implementation of tailored preventive and therapeutic strategies for this high-risk population.

**Disclosure of Interest:**

None Declared

## Long descriptions

### Table 1. Long description

The table consists of five columns: Outcome, S U D (N equals 69,373), non-S U D (N equals 69,373), Hazard ratio (95 percent C I), and p value.

* A M I: S U D group has 657 cases, non-S U D has 263. Hazard ratio is 2.916 (2.528, 3.365) with a p value less than .001.

* H F: S U D group has 1,256 cases, non-S U D has 548. Hazard ratio is 2.685 (2.428, 2.968) with a p value less than .001.

* A F: S U D group has 537 cases, non-S U D has 314. Hazard ratio is 1.994 (1.735, 2.292) with a p value less than .001.

* Mortality: S U D group has 4,074 cases, non-S U D has 2,836. Hazard ratio is 1.621 (1.546, 1.701) with a p value less than .001.

All p values are marked with an asterisk indicating statistical significance. Initialisms used: S U D (Substance use disorder), A M I (Acute myocardial infarction), A F (Atrial fibrillation), H F (Heart failure), H R (Hazard ratio), and C I (Confidence interval).

Navigate back to Table 1..

### Image 1: Long description

The flowchart begins at the top with a box for patients newly diagnosed with T 2 D M between January 1st 2016 and December 31st 2022, aged 18 years or older.

Below this, a horizontal line branches to the right to an exclusion box: Exclude preexisting conditions of A M I, A F, or H F.

The main path continues down and splits into two primary cohorts:

1. Left: S U D cohort (n equals 70,179) defined by opioid, cannabis, or cocaine use disorders within one year prior to T 2 D M diagnosis.

2. Right: Non-S U D cohort (n equals 2,122,587) with no documented diagnosis of opioid, cannabis, or cocaine use disorders.

Arrows from both cohorts point inward to a central box: 1 to 1 P S M by age, demographics and lifestyle, underlying diseases and medication.

Following the P S M process, the cohorts are refined to equal sizes:

1. Left: S U D cohort (n equals 69,373).

2. Right: Non-S U D cohort (n equals 69,373).

Both final cohort boxes point down to a terminal box at the bottom: Whether the patient was newly diagnosed A M I, A F, and H F during a 5-year-follow-up.

Navigate back to Image 1:.

### Image 2: Long description

A four-panel set of line graphs. Each graph shares the same axes: the x-axis represents Time in days from 0 to 1000, and the y-axis represents Survival probability from 0.92 to 1.00. Two groups are plotted in each: a red line for the S U D cohort and a teal line for the non-S U D cohort. All panels include the statistical note p < 0.001.

* Panel A. A M I: Both cohorts start at 1.00. The teal line remains nearly flat, ending just below 1.00. The red line shows a gradual downward curve, ending at approximately 0.985.

* Panel B. H F: Both cohorts start at 1.00. The teal line declines slightly to 0.99. The red line shows a more pronounced downward curve, ending at approximately 0.975.

* Panel C. A F: Both cohorts start at 1.00. The teal line ends near 0.995. The red line curves downward to end at approximately 0.99.

* Panel D. Mortality: This panel shows the steepest decline for both groups. Both start at 1.00. The teal line follows a linear-like decrease to 0.955. The red line drops more sharply in a concave curve, ending at approximately 0.925.

Navigate back to Image 2:.

### Image 3: Long description

The table and forest plot are organized into three main sections based on substance use. The x-axis of the forest plot represents the Hazard Ratio, abbreviated as H R, ranging from 1 to 4.5, with a vertical red line at 1 indicating the null effect.

1. Cannabis section

- A M I: H R 2.955, 95 percent C I [2.434, 3.587], p-value less than 0.001.

- A F: H R 2.651, 95 percent C I [2.141, 3.282], p-value less than 0.001.

- H F: H R 2.928, 95 percent C I [2.534, 3.383], p-value less than 0.001.

- Mortality: H R 1.547, 95 percent C I [1.442, 1.660], p-value less than 0.001.

2. Cocaine section

- A M I: H R 3.383, 95 percent C I [2.501, 4.575], p-value less than 0.001.

- A F: H R 2.327, 95 percent C I [1.696, 3.192], p-value less than 0.001.

- H F: H R 3.497, 95 percent C I [2.811, 4.351], p-value less than 0.001.

- Mortality: H R 1.515, 95 percent C I [1.375, 1.670], p-value less than 0.001.

3. Opioid section

- A M I: H R 3.020, 95 percent C I [2.429, 3.755], p-value less than 0.001.

- A F: H R 2.379, 95 percent C I [1.936, 2.923], p-value less than 0.001.

- H F: H R 2.739, 95 percent C I [2.371, 3.164], p-value less than 0.001.

- Mortality: H R 1.804, 95 percent C I [1.690, 1.926], p-value less than 0.001.

In all cases, the blue square markers and their horizontal error bars are positioned entirely to the right of the red line at 1, indicating a statistically significant increased risk for all outcomes across all three substances.

Navigate back to Image 3:.

